# Anatomical recovery of the spinal glutamatergic system following a complete spinal cord injury in lampreys

**DOI:** 10.1038/srep37786

**Published:** 2016-11-25

**Authors:** Blanca Fernández-López, Antón Barreiro-Iglesias, María Celina Rodicio

**Affiliations:** 1 Department of Functional Biology, CIBUS, Faculty of Biology, Universidade de Santiago de Compostela, 15782, Santiago de Compostela, Spain

## Abstract

Lampreys recover locomotion following a spinal cord injury (SCI). Glutamate is necessary to initiate and control locomotion and recent data suggest a crucial role for intraspinal neurons in functional recovery following SCI. We aimed to determine whether, in lampreys, axotomized spinal glutamatergic neurons, which lose glutamate immunoreactivity immediately after SCI, recover it later on and to study the long-term evolution and anatomical recovery of the spinal glutamatergic system after SCI. We used glutamate immunoreactivity to study changes in the glutamatergic system, tract-tracing to label axotomized neurons and TUNEL labelling to study cell death. Transections of the cord were made at the level of the fifth gill. TUNEL experiments indicated that cell death is a minor contributor to the initial loss of glutamate immunoreactivity. At least some of the axotomized neurons lose glutamate immunoreactivity, survive and recover glutamate immunoreactivity 1 week post-lesion (wpl). We observed a progressive increase in the number of glutamatergic neurons/processes until an almost complete anatomical recovery at 10 wpl. Among all the glutamatergic populations, the population of cerebrospinal fluid-contacting cells is the only one that never recovers. Our results indicate that full recovery of the glutamatergic system is not necessary for the restoration of function in lampreys.

In mammals, including humans, spinal cord injury (SCI) leads to permanent disability and to an irreversible loss of movement and sensitivity below the site of lesion. This has become an important health and economic problem due to the lack of adequate treatment. In contrast to mammals, most anamniote vertebrates are capable of spinal cord regeneration and functional recovery after SCI^1^. Lampreys, together with jawed fishes, are the only vertebrates that satisfy the five criteria established by the US National Institutes of Health (NIH) for considering complete functional regeneration [(1) The experimental lesion must cause disconnection of nerve processes, (2) processes of CNS neurons must bridge the level of injury, (3) the regenerated fibres must make junctional contacts, (4) the regenerated fibres must generate post-junctional responses and (5) changes in function must derive from regenerated connections]^2^,^3^; therefore, they have become a vertebrate model for the study of spontaneous spinal cord regeneration since the 1950 s^4^.

Functional recovery after SCI requires both the regeneration of lost neurons and damaged axons and the formation of new synaptic contacts. Restoration of function after SCI has been mainly related to the regeneration of descending axons from spinal-projecting neurons. However, data from different models indicating a crucial role for intraspinal neurons in achieving functional recovery has been increasingly larger^5^,^6^,^7^,^8^,^9^,^10^; reviewed in ref. 11, including work in lampreys^12^,^13^,^14^,^15^. In lampreys, brain spinal-projecting^16^,^17^ and intrinsic spinal cord neurons can regenerate their axons across the lesion site^12^,^13^,^14^, and the regenerated axons are able to establish synaptic contacts below the site^15^. It has been suggested that intrinsic spinal neurons relay descending drive to locomotor networks in more caudal regions after SCI in lampreys, since the function at a certain spinal level is restored before the descending reticulospinal axons reach that level of the cord^13^,^16^. This indicates that the role of intraspinal neurons becomes even more important after SCI than under normal conditions. As not all damaged axons regenerate, even in lampreys^17^, there is increasing interest in determining the compensatory plastic changes that must take place in the locomotor networks above and below the site of lesion for the recovery of locomotor function^18^.

Glutamate mediates excitatory neurotransmission and is used by neurons in the command centres (diencephalic and mesencephalic locomotor regions), reticulospinal neurons and neurons in the spinal cord that excite spinal motor neurons and interneurons^19^,^20^. Excessive glutamate release in response to a lesion causes glutamate excitotoxicity, which can lead to neuronal and oligodendrocyte death^21^,^22^,^23^, thus worsening the outcome of the lesion. While the short term changes in glutamate levels after SCI are well documented^21^,^24^, even in lampreys^25^, as well as the changes of other neurotransmitter systems during regeneration in lampreys or zebrafish (for example, the serotonergic system)^26^,^27^,^28^,^29^; surprisingly, much less is known about the long term changes in the glutamatergic system.

In a recent study in lampreys, we found a complete disappearance of glutamate immunoreactivity immediately after a complete transection of the cord rostrally and caudally to the site of injury^25^, which was related to excessive glutamate release. Based on these previous results, the main aims of the present study were (1) to determine whether, in lampreys, axotomized glutamatergic neurons that lose glutamate immunoreactivity immediately after SCI are able to recover it later on, evaluating the possible contribution of cell death to this initial loss of glutamate immunoreactivity and (2) to study the long-term evolution of the glutamatergic system (cells and fibres) after a complete SCI in lampreys. Identifying the changes that occur in the spinal cord during spontaneous recovery of locomotion in regenerating vertebrates may offer clues to determine the key events that lead to the recovery of function as well as to decipher the overall mechanisms of plasticity that take place during the regenerative process.

## Results

### Glutamate immunoreactivity in the spinal cord of un-lesioned animals

**Figure 1:**
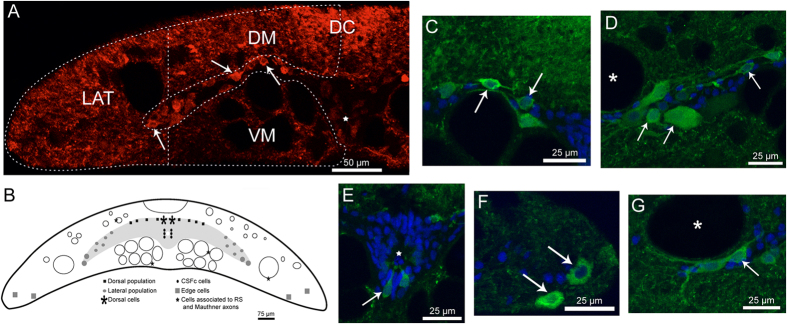
Glutamate immunoreactivity in the spinal cord at the level of the 5^th^ gill in a control un-lesioned sea lamprey. (**A**) Confocal photomicrograph of a transverse section of the spinal cord showing the 3 regions in which the white matter was divided to quantify the number of glutamate immunoreactive (-ir) profiles. Dorsomedial (DM), ventromedial (VM) and lateral (LAT). DC indicates the dorsal column. (**B**) Schematic drawing of a transverse section of the spinal cord showing the glutamate-ir cell types in the larval spinal cord. (**C–G**) High magnification photomicrographs showing details of glutamate-ir cells (green) and nuclear counterstain (blue). (**C**) Cells of the dorsal population. (**D**) Cells of the lateral population. (**E**) Cerebrospinal fluid-contacting (CSFc) cells. (**F**) Edge cells. (**G**) Cells associated to the Mauthner axon. Arrows point to glutamate-ir cells. Asterisks point to Mauthner axon. Star indicates the central canal. In all photomicrographs, dorsal is at the top and the midline to the right, except for (**F**), in which the midline is on the left.

This work is focused on the study of the changes in the glutamatergic system following a complete SCI in the sea lamprey. A detailed description of the spinal glutamatergic system of the sea lamprey has been previously reported^30^. Briefly, in control un-lesioned animals, glutamate immunoreactive (-ir) neuronal processes were observed in the dorsal, ventral and lateral columns of the white matter, being more abundant in the dorsal column (Fig. 1A). The grey matter forms paired “horns” extending laterally. The dorsomedial region corresponds to the dorsal horns of the spinal cord of jawed vertebrates and the lateral region to the ventral horns. Two main populations of glutamatergic cells were observed in the grey matter [the dorsal (Fig. 1C) and lateral (Fig. 1D) populations], together with the cerebrospinal fluid-contacting (CSFc) cells (Fig. 1B,E) and the primary sensory dorsal cells (Fig. 1B). Individual cells were also observed in the white matter [edge cells (Fig. 1F) and cells associated with Mauthner (Fig. 1G), Müller and medium-sized dorsolateral axons (Fig. 1B)]. The dorsal cells and the cells of the white matter did not appear in all spinal cord sections of control un-lesioned animals, with some of them being observed only occasionally, therefore, we did not include these cells in the quantifications to avoid bias.

### Cell death cannot explain the complete disappearance of glutamate immunoreactivity after the lesion

**Figure 2:**
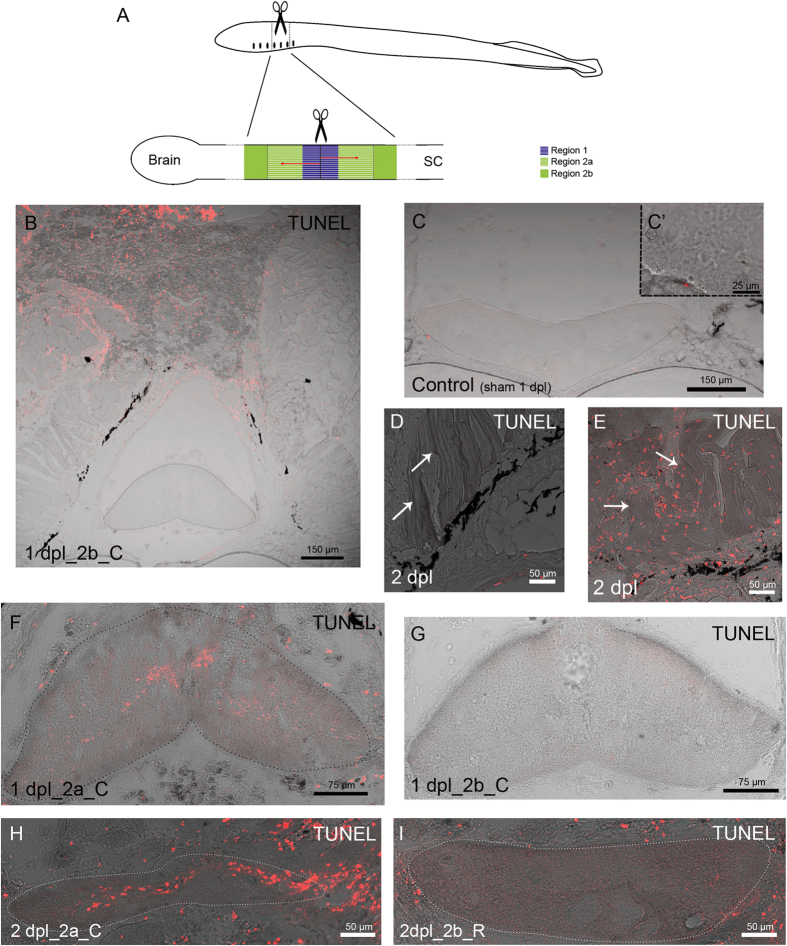
Cell death in the spinal cord following the complete spinal cord transection. (**A**) Schematic representations of a lateral view of a larva indicating the site of injury (scissors) and a lateral view of the spinal cord (SC) indicating the regions of analysis and how axotomized glutamate-ir neurons are labelled by the tracer (red). (**B–I**) Bright-field images merged with confocal images showing TUNEL staining in control (**C**), 1 day post-lesion (dpl) (**B,F,G**) and 2 dpl (**D,E,H,I**) larvae. (**B**) General view of the caudal (**C**) spinal cord (region 2b) and the surrounding tissue in a 1 dpl animal. Note the presence of TUNEL labelling in the skin and muscle damaged by the injury but not in the spinal cord. (**C**) Transverse section of the spinal cord of a sham control larvae showing no TUNEL labelling. Note the presence of a TUNEL-stained nucleus in the meninx. (**C**’) High-magnification photomicrograph showing the detail of the TUNEL-stained nucleus in the meninx. (**D**) Absence of TUNEL staining in undamaged muscle fibres. (**E**) TUNEL staining in damaged muscle fibres (arrows). (**F**) Transverse section of the region 2a of the spinal cord showing the presence of cell death at 1 dpl in the caudal stump. (**G**) Transverse section of the region 2b of the spinal cord showing absence of cell death at 1 dpl in the caudal stump. (**H**) Transverse section of the region 2a of the spinal cord showing the presence of cell death, caudally to the site of injury, at 2 dpl. (**I**) Transverse section showing the absence of cell death in region 2b of the rostral stump of the spinal cordat 2 dpl. Note that there are some nuclei labelled by TUNEL in the surrounding tissue. Dotted lines delineate the spinal cord in (**B**,**C**,**F**,**H** and **I**).

For a better understanding of the present results we divided the rostral and caudal spinal cord near the site of injury in different regions: region 1, which corresponds to the first 150 μm rostral and caudal from the epicentre of the injury and region 2, which corresponds to the next 500 μm rostral and caudal to the site of injury (Fig. 2A). Region 1 is completely damaged due to the mechanical lesion, therefore, region 2 was the area analyzed in the present study.

In a previous study from our group we observed the complete loss of glutamate immunoreactivity in region 2 of the spinal cord immediately after a complete SCI^25^. Here, we used TUNEL labelling at 1 and 2 days post-lesion (dpl) to reveal a possible contribution of cell death to this initial loss of glutamate immunoreactivity (Fig. 2). Based on the presence or absence of TUNEL labelling, region 2 was subdivided in regions 2a (first 300 μm of region 2 from the site of injury) and 2b (next 200 μm) (Fig. 2A). Sham-operated control animals did not show TUNEL labelling in the spinal cord (Fig. 2C,C’). The musculature of the body wall at the site of lesion served as a good internal control (positive and negative) for the TUNEL experiment. TUNEL labelling was only observed in the damaged muscle fibres (Fig. 2E), while no TUNEL labelling was observed in more lateral muscles that were not damaged during the surgical procedures (Fig. 2D). In the spinal cord, TUNEL labelled cells were observed in transverse sections of regions 1 and 2a of the spinal cord at 1 and 2 dpl (Fig. 2F,H). However, we did not observe any TUNEL labelled cell in sections of region 2b of the spinal cord at 1 and 2 dpl (Fig. 2B,G,I). This indicates that cell death is not the cause of the disappearance of glutamate immunoreactivity in region 2b of the spinal cord following a complete SCI; although, cell death could still contribute to the disappearance of glutamate immunoreactivity in region 2a.

### Tract-tracing experiments indicate that at least some of the axotomized glutamatergic neurons of region 2a do not die and are able to recover glutamate immunoreactivity

**Figure 3:**
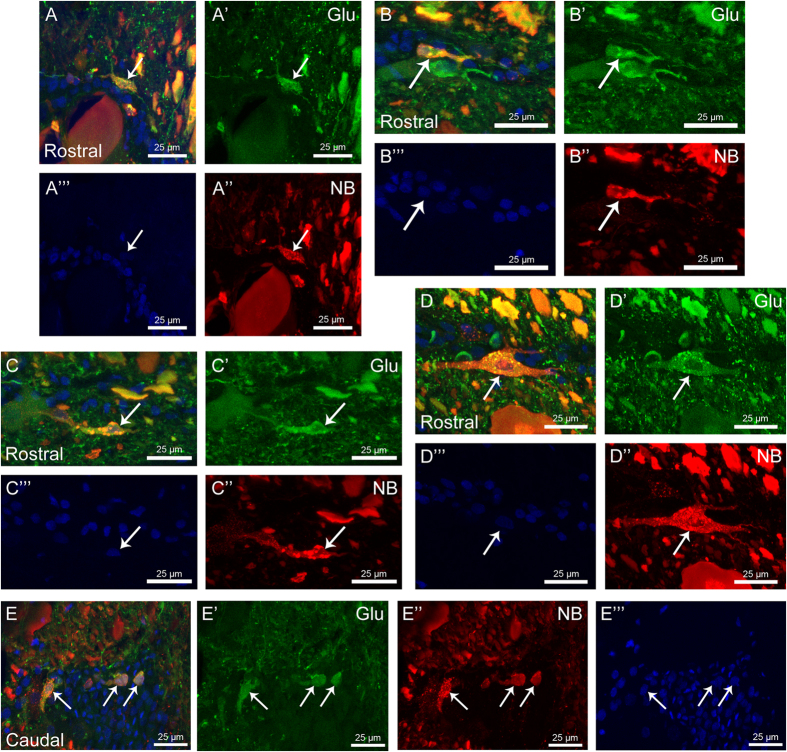
Recovery of glutamate immunoreactivity in axotomized glutamatergic cells. High magnification confocal photomicrographs of transverse sections of the rostral (**A–D**”) and caudal (**E–E**”) spinal cord of 1wpl larvae showing double immunolabelled cells (arrows) for glutamate and Neurobiotin. (**A–A**”’) Cell of the dorsal population. (**B–B**”’) Small cell of the lateral population. (**C–C**”’) Edge cell. (**D–D**”’) Large cell of the lateral population. (**E–E**”’) Cells of the lateral population. Dorsal is at the top. The midline is to the left, except for (**E–E**”’), in which the midline is to the right. (**A–E**) Overlay; (**A**’–**E**’) Glutamate; (**A**”–**E**”) Neurobiotin; (**A**”–**E**”’) Nuclear staining.

In the tract-tracing experiments, the tracer Neurobiotin was applied in both stumps of the spinal cord at the time of the complete spinal cord transection. The animals were processed at 1 and 2 weeks post-lesion (wpl) and region 2a was analysed. The time-points were chosen based on our previous study^25^, in which glutamate immunoreactivity in the spinal cord was recovered in the first 500 μm rostral and caudal from the site of injury at 1 wpl. Rostral to the site of injury, 45% of the glutamate-ir cells of the grey matter glutamatergic populations (Fig. 3A-B”’, D–D”’) and 60% of the glutamate-ir cells of the white matter (Fig. 3C–C”’) showed glutamate and neurobiotin colocalization at 1 wpl. Caudally, 43% of the glutamate-ir cells of the grey matter populations (Fig. 3E–E”’) and 50% of the glutamate-ir cells of the white matter showed glutamate and neurobiotin colocalization at 1 wpl. Similar rates of colocalization were observed at 2 wpl. This indicates that, even in region 2a, where some cell death is observed at 1 and 2 dpl (see above), at least some damaged glutamatergic neurons lose glutamate immunoreactivity, survive and later on recover glutamate immunoreactivity.

### Long-term recovery of glutamatergic cell numbers during spinal cord regeneration

**Figure 4:**
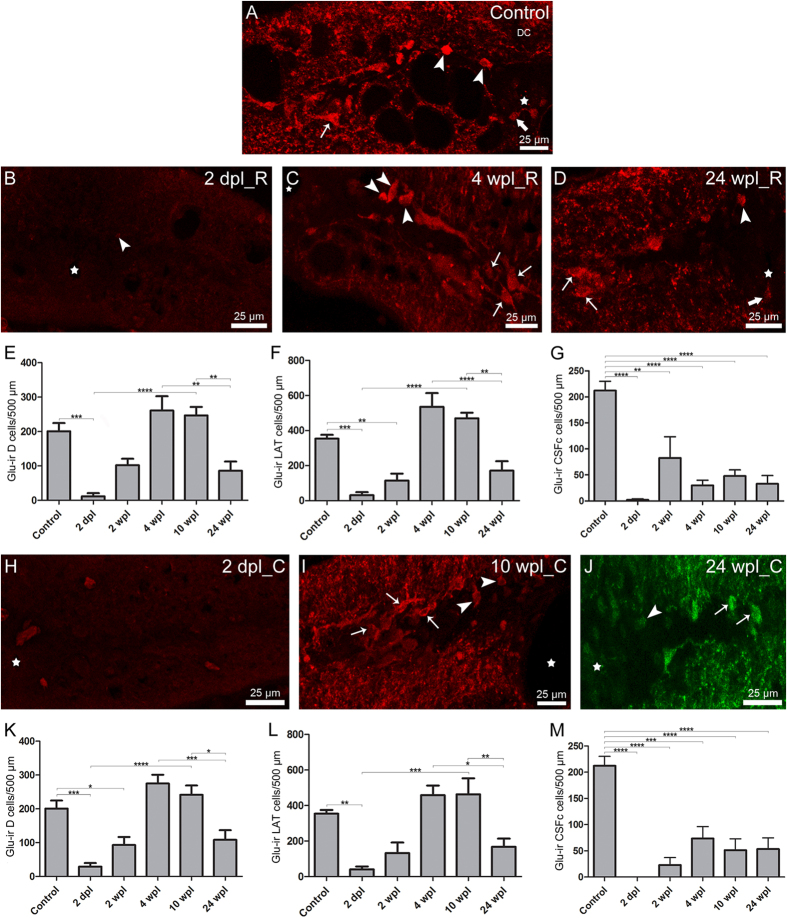
Long-term changes in glutamatergic cell numbers. (**A–D**) Photomicrographs of transverse sections of the spinal cord, showing glutamate-ir cells in control larvae (**A**) and in the rostral (**R**) stump of 2 dpl (**B**), 4 wpl(**C**) and 24 wpl (**D**) larvae. Dorsal interneurons (arrowheads); lateral interneurons (arrows); CSFc cells (thick arrows); central canal (star). (**E–G**) Graphs showing the number of glutamate-ir dorsal interneurons (**E**), lateral interneurons (**F**) and CSFc cells (**G**) in the rostral stump of the spinal cord. (**E**) Control: 200.8 ± 23.6; 2 dpl: 11.4 ± 9.3; 2 wpl: 102.1 ± 18.9; 4 wpl: 260.9 ± 41.6; 10 wpl: 246.9 ± 24.6; 24 wpl: 86.3 ± 26.5. ANOVA, *p* < 0.0001. (**F**) Control: 353.4 ± 21.8; 2 dpl: 31.3 ± 16.8; 2 wpl: 115.3 ± 39.2; 4 wpl: 535.0 ± 78.9; 10 wpl: 469.9 ± 31.7; 24 wpl: 170.9 ± 53.9. ANOVA, *p* < 0.0001. (**G**) Control: 212.3 ± 17.7; 2 dpl: 2.0 ± 2.0; 2 wpl: 82.5 ± 40.9; 4 wpl: 29.9 ± 9.9; 10 wpl: 47.8 ± 11.8; 24 wpl: 32.8 ± 16.1. ANOVA, *p* < 0.0001. (**H–J**) Photomicrographs of transverse sections of the spinal cord, showing glutamate-ir cells in the caudal (**C**) stump of 2 dpl (**H**), 10 wpl (**I**) and 24 wpl (**J**) larvae. (**K–M**) Graphs showing the number of glutamate-ir dorsal interneurons (**K**), lateral interneurons (**L**) and CSFc cells (**M**) in the caudal stump of the spinal cord. (**K**) Control: 200.8 ± 23.6; 2 dpl: 29.3 ± 10.2; 2 wpl: 93.2 ± 23.7; 4 wpl: 274.9 ± 25.9; 10 wpl: 241.3 ± 27.8; 24 wpl: 108.7 ± 28.1. ANOVA, *p* < 0.0001. (**L**) Control: 353.4 ± 21.8; 2 dpl: 40.2 ± 16.9; 2 wpl: 132.8 ± 58.6; 4 wpl: 458.1 ± 54.3; 10 wpl: 463.1 ± 89.9; 24 wpl: 167.6 ± 45.9. ANOVA, *p* < 0.0001. (**M**) Control: 212.3 ± 17.7; 2 dpl: 0.0 ± 0.0; 2 wpl: 23.0 ± 14.1; 4 wpl: 73.7 ± 22.5; 10 wpl: 51.4 ± 21.7; 24 wpl: 53.2 ± 21.6. ANOVA, *p* < 0.0001. *0.01–0.05; **0.01–0.001; ***0.001–0.0001; **** < 0.0001.

The changes in glutamate-ir cell numbers at different time points after the injury were analysed in region 2 of the spinal cord and are shown in Fig. 4. In injured animals, the general distribution of the different cell populations was very similar to that of the un-lesioned animals, with no remarkable changes. Rostral to the site of injury, we observed a significant 94.3% reduction in the number of glutamate-ir neurons of the dorsal population at 2 dpl, as compared to control un-lesioned animals. At 10 wpl, the number of cells of the dorsal population was not significantly different to control animals. At 24 wpl, the number of cells of the dorsal population was significantly reduced by 66.9% and 65% as compared to 4 and 10 wpl, respectively. No significant differences were found in the number of cells of the dorsal population between 24 wpl and control animals (Fig. 4A–E). Caudal to the site of injury, at 2 dpl and 2 wpl we found a significant 85.4% and 53.6% reduction, respectively, in the number of cells of the dorsal population as compared to control un-lesioned animals. At 10 wpl, the number of cells of the dorsal population was not significantly different to control animals. At 24 wpl, the number of cells of the dorsal population was significantly reduced by 60.4% and 54.9% as compared to 4 and 10 wpl, respectively. No significant differences were found between control and 24 wpl animals (Fig. 4A,H–K).

Rostral to the site of injury, we observed a significant 91.1% and 67.4% reduction in the number of cells of the lateral population at 2 dpl and 2 wpl, respectively, as compared to control un-lesioned animals. At 10 wpl the number of cells of the lateral population was not significantly different to control animals. At 24 wpl, the number of cells of the dorsal population was significantly reduced by 68% and 63.6% as compared to 4 and 10 wpl, respectively. No significant differences were found in the number of cells of the lateral population between 24 wpl and control animals (Fig. 4F). Caudal to the site of injury, at 2 dpl we found a significant 88.6% reduction in the number of cells of the lateral population as compared to un-lesioned animals. At 10 wpl, the number of cells of the lateral population was not significantly different to control animals. At 24 wpl, the number of cells of the lateral population was significantly reduced by 63.4% and 63.8% as compared to 4 and 10 wpl, respectively and was not significantly different to un-lesioned animals (Fig. 4L).

Rostral and caudal to the site of injury, the number of glutamate-ir CSFc cells was significantly reduced even at 24 wpl. Significant differences between control animals and each experimental group were found. Rostral to the site of injury, at 2 dpl and 2, 4, 10 and 24 wpl, we observed a 99.1%, 61.1%, 85.9%, 77.5% and 84.5% reduction in the number of CSFc cells, respectively, as compared to control un-lesioned animals (Fig. 4G). Caudal to the site of injury, the number of CSFc cells was significantly reduced by 100%, 89.2%, 65.3%, 75.8% and 74.9% at 2 dpl, 2, 4, 10 and 24 wpl, respectively, as compared to control animals (Fig. 4M).

### Quantitative changes in the number of glutamate immunoreactive processes during spinal cord regeneration

**Figure 5:**
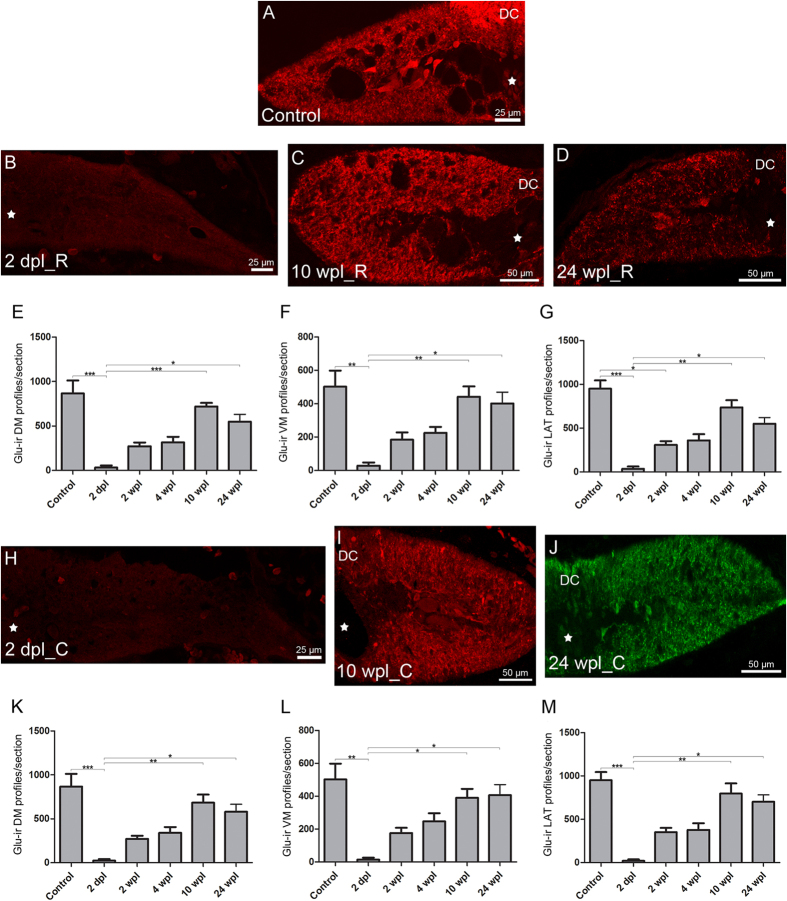
Long-term changes in the number of glutamate-ir profiles. (**A–D**) Photomicrographs of transverse sections of the spinal cord, showing glutamate-ir processes in control larvae (**A**) and in the rostral stump of 2 dpl (**B**), 10 wpl (**C**) and 24 wpl (**D**) larvae. Central canal (star). (**E–G**) Graphs showing the number of glutamate-ir dorsomedial (**E**), ventromedial (**F**) and lateral (**G**) profiles in the rostral stump of the spinal cord. (**E**) Control: 866.5 ± 145.8; 2 dpl: 32.7 ± 23.1; 2 wpl: 273.3 ± 41.4; 4 wpl: 315.6 ± 62.5; 10 wpl: 719.1 ± 40.4; 24 wpl: 549.2 ± 82.5. Kruskal-Wallis, *p* = 0.0002. (**F**) Control: 502.1 ± 96.1; 2 dpl: 29.0 ± 19.5; 2 wpl: 184.6 ± 43.7; 4 wpl: 224.7 ± 36.6; 10 wpl: 442.0 ± 61.7; 24 wpl: 401.5 ± 67.8. Kruskal-Wallis, *p* = 0.0009. (**G**) Control: 950.5 ± 94.8; 2 dpl: 35.3 ± 26.7; 2 wpl: 309.1 ± 41.7; 4 wpl: 360.2 ± 72.3; 10 wpl: 738.4 ± 81.2; 24 wpl: 550.7 ± 70.8. Kruskal-Wallis, *p* = 0.0002. (**H–J**) Photomicrographs of transverse sections of the spinal cord, showing glutamate-ir processes in the caudal stump of 2 dpl (**H**), 10 wpl (**I**) and 24 wpl (**J**) larvae. (**K–M**) Graphs showing the number of glutamate-ir dorsomedial (**K**), ventromedial (**L**) and lateral (**M**) profiles in the caudal stump of the spinal cord. (**K**) Control: 866.5 ± 145.8; 2 dpl: 25.9 ± 15.7; 2 wpl: 270.1 ± 36.8; 4 wpl: 341.1 ± 65.2; 10 wpl: 684.4 ± 93.0; 24 wpl: 580.1 ± 86.4. Kruskal-Wallis, *p* = 0.0003. (**L**) Control: 502.1 ± 96.1; 2 dpl: 14.6 ± 11.9; 2 wpl: 176.6 ± 31.5; 4 wpl: 247.5 ± 49.6; 10 wpl: 390.6 ± 54.1; 24 wpl: 406.9 ± 63.8. Kruskal-Wallis, *p* = 0.0013. (**M**) Control: 950.5 ± 94.8; 2 dpl: 20.8 ± 15.8; 2 wpl: 350.4 ± 50.1; 4 wpl: 377.4 ± 76.9; 10 wpl: 798.4 ± 115.6; 24 wpl: 702.8 ± 79.6. Kruskal-Wallis, *p* = 0.0002. *0.01–0.05; **0.01–0.001; ***0.001–0.0001; **** < 0.0001.

Variation in the number of glutamate-ir profiles was studied at 2 dpl and at 2, 4, 10 and 24 wpl, as these time points correspond to different stages during the regeneration of descending axons. During the first 2 wpl, axonal retraction predominates with retractions of up to 2 mm^12^,^31^,^32^. The descending axons then start to grow and reach the rostral stump at 4 wpl; between 5 and 7 wpl the descending axons enter the caudal stump^12^. At 10 wpl, most of the regenerating axons may be re-innervating the caudal stump and at 24 wpl the regeneration could be considered complete. Normal appearing swimming behaviour can already be observed at 10 wpl. The changes in the number of glutamate-ir profiles are shown in Fig. 5. In the rostral dorsomedial (DM) region, we observed a significant 96.2% reduction in the number of glutamate-ir profiles at 2 dpl as compared to control un-lesioned animals. At 10 and 24 wpl the number of glutamate-ir profiles was not significantly different to control animals (Fig. 5A–E). Caudal to the site of injury, at 2 dpl we observed a significant 97% decrease in the number of glutamate-ir profiles in the DM region. At 10 and 24 wpl the number of glutamate-ir profiles was not significantly different to control animals (Fig. 5A,H–K).

In the rostral ventromedial (VM) region, we found a significant 94.2% reduction in the number of glutamate-ir profiles at 2 dpl as compared to control un-lesioned animals. At 10 and 24 wpl, the number of glutamate-ir profiles was not significantly different to control un-lesioned animals (Fig. 5F). Caudal to the site of injury, the number of glutamate-ir profiles in the VM region was significantly reduced by 97.1% at 2 dpl as compared to control un-lesioned animals. At 10 and 24 wpl, the number of glutamate-ir profiles was not significantly different to control un-lesioned animals (Fig. 5L).

Rostral to the site of injury, we found a significant 96.3% and 67.5% reduction in the number of glutamate-ir profiles at 2 dpl and 2 wpl, respectively, in the lateral (LAT) region of the cord as compared to control animals. At 10 and 24 wpl, the number of glutamate-ir profiles was not significantly different to control un-lesioned animals (Fig. 5G). Caudal to the site of injury, the number of glutamate-ir profiles was significantly reduced by 97.8% in the LAT region at 2 dpl as compared to control un-lesioned animals. At 10 and 24 wpl, the number of glutamate-ir profiles was not significantly different to control un-lesioned animals (Fig. 5M).

## Discussion

The present study shows the changes that occur in the spinal glutamatergic system of the sea lamprey during recovery following a complete SCI. Despite the importance of glutamatergic signalling in the circuits underlying locomotion (for a review, see ref. 33), changes in this system during spinal cord regeneration have not been previously investigated. We show that, in lampreys, the drastic initial decrease in the number of glutamate-ir cells and processes in response to a complete spinal cord lesion is followed by an almost complete recovery of the system; with the exception of the CSFc cells that are never recovered, even at 24 wpl. This is in contrast with the dopaminergic system, which undergoes full anatomical recovery following a complete injury at the same spinal cord level in lampreys^34^. However, the serotonergic system of lampreys also shows an incomplete recovery after SCI^26^.

Regarding changes in the number of glutamatergic cells, we show that in the dorsal and lateral populations (rostral and caudal to the site of injury) there is a drastic reduction (>85%) in cell numbers at 2 dpl. This is in agreement with our previous results in which we have reported a complete disappearance of glutamate immunoreactivity in region 2 of the spinal cord immediately after a SCI^25^. This is followed by a gradual increase until 4 to 10 wpl, when the number of glutamate-ir cells is slightly higher than that of control un-lesioned animals (between 1.2 to1.5 times higher). At 24 wpl, the number of cells in the two populations returned to control levels due to a significant decrease in the number of cells as compared to 4 and 10 wpl. The TUNEL and tract-tracing experiments indicated that probably cell death is not the main cause of the initial disappearance of glutamate-ir cells, thus suggesting that it could mainly be due to glutamate release from the neurons. The presence of double-labelled neurobiotin/glutamate-ir cells at 1 and 2 wpl demonstrates that, in lampreys, around 40–45% of the cells of the dorsal and lateral populations and 50% of the cells of the white matter are axotomized during the injury, release glutamate, survive, and recover their glutamate content during the recovery process. As far as we are aware, this is the first time that this phenomenon is directly observed in vertebrates by using histological and immunohistochemical methods. It is also noteworthy the fact that glutamate immunoreactivity could be lost immediately after the injury not only in axotomized neurons but also in non-axotomized neurons, as it is suggested by the presence of glutamate-ir neurons at 1 wpl that are not labelled by neurobiotin. However, this cannot be assured since the efficiency of Neurobiotin labelling migth not be 100%.

A contribution of cell death to the initial loss of glutamatergic cells in region 2a is also possible. The disappearance of glutamate immunoreactivity immediately after the injury has precluded us from assessing the glutamatergic phenotype of dying cells. If some glutamatergic neurons die in region 2a, the full recovery of glutamatergic cells in the dorsal and lateral populations would require the generation of new glutamatergic neurons and/or a change in neurotransmitter phenotype of surviving non-glutamatergic neurons. These processes must be probably happening since we have observed a slight increase in the number of cells in these populations at 4 and 10 wpl. Production of new neurons after SCI in lampreys has been recently reported^35^, although the neurotransmitter phenotype of these new neurons has not been determined. Taken together, present and previous results suggest that glutamatergic interneurons could be newly generated after the injury in lampreys. Due to differences in the fixation methods, double staining with the proliferation markers available in lampreys and the anti-glutamate antibodies was not carried out. A similar overproduction response has been previously reported for the generation of new motor neurons^36^ and serotonergic neurons^29^ after a spinal lesion in zebrafish. However, neuronal overproduction after SCI in zebrafish is much larger, up to 5-fold the number of neurons in control animals^29^. By contrast, the number of dopaminergic neurons after SCI in lampreys increases progressively until reaching levels similar to those found in un-lesioned animals, but without an initial phase of overproduction of dopaminergic neurons^34^. This highlights the importance of studying the behaviour of neurons with different neurotransmitter phenotypes to decipher how they respond to the injury and their contribution to functional restoration after a lesion.

In contrast to the dorsal and lateral populations, a large decrease in the number of glutamate-ir CSFc cells was still observed in both stumps of the spinal cord at time points in which functional recovery is already achieved (10 to 24 wpl)^37^. In addition to cell death and glutamate release to the extracellular space, glutamate release to the CSF may account for the decrease in the number of glutamate-ir CSFc cells. Long lasting high glutamate levels in the CSF have been reported in subjects with amyotrophic lateral sclerosis^38^ and motor neuron disease^39^. However, the relevance of this phenomenon is not well understood and further studies are necessary to clarify this issue.

From 2 to 4 and 10 wpl, the number of glutamate-ir processes increases continuously, reaching numbers similar to control animals in the different spinal cord regions. This fits with the regeneration of the reticulospinal (RS) axons and with electrophysiological studies, since at 8 wpl locomotor activity is observed up to mid spinal cord levels and spinal networks at this level can be activated by restored descending RS neurons^40^.

Quantification of the glutamate-ir neuronal populations and processes during recovery from SCI has showed slight differences between the rostral and caudal stumps. Although small, these differences could have important functional implications. The dorsal population of glutamate-ir cells recovers faster in the rostral than in the caudal stump (2 vs 4 wpl, respectively). On the other hand, the lateral population of glutamate-ir cells recovers faster in the caudal than in the rostral stump (2 vs 4 wpl, respectively). Cells of the lateral population are considered to be mainly excitatory interneurons (EINs) that participate in the central pattern generator (CPG)^30^, excite motor neurons and other interneurons and are involved in rhythm generation (for review see ref. 19,20). Cells of the dorsal population probably participate in the sensory relay and have dendrites that reach the dorsal column^30^. In electrophysiological studies, muscle locomotor activity has been observed at 2 wpl just below the lesion^16^,^40^. Descending RS axons suffer a retraction during the first 2 wpl, reach the rostral stump at 4 wpl and the caudal stump at 5–7 wpl^12^. So, the muscle locomotor activity observed at 2 wpl has to be attributed to the propriospinal network, which is in agreement with the recovery of the lateral population at 2 wpl in the caudal stump (present study). A reorganization of the spinal circuits could occur to relay the descending drive to more caudal regions of the cord. Since the dorsal population recovers rostrally at 2 wpl, these cells could be participating in the locomotor relay following injury, interacting with the EINs of the caudal stump. The idea of propiospinal cells relaying the drive to locomotor networks in more caudal areas of the spinal cord after an injury in lampreys has been previously suggested^13^, but the cells responsible for this process have not been indetified. Propriospinal relay connections can bypass the site of injury mediating spontaneous functional recovery and supraspinal control of locomotion in mammals^7^,^9^,^10^, which suggest that this phenomenon is higly conserved in vertebrates, including lampreys and mammals.

The results analysing the changes in the number of glutamate-ir processes also support the idea of cells of the dorsal population relaying the descending drive through the site of injury to the cells of the lateral population of the caudal stump. The number of processes of the lateral rostral stump is recovered at 4 wpl, whereas in the caudal stump there are no significant differences with control un-lesioned animals at 2 wpl. The number of processes in the dorsomedial region is recovered at 2 wpl both rostral and caudal to the site of injury. Most of the processes of the lateral region correspond to small RS glutamatergic neurons of several rhombencephalic nuclei (anterior, middle and posterior rhombencephalic reticular nuclei^41^ and the postero-lateral vagal group^42^) and to processes of the cells of the lateral population of the spinal cord. Based on the know timing of regeneration of descending neurons (see above), the recovery of the number of processes at 2 wpl in the caudal stump has to be attributed to the intrinsic lateral spinal neurons. In the rostral stump, the number of processes of the lateral region recovers at 4 wpl, which is when RS axons are reinnervating this stump of the cord. Processes of the dorsomedial region comprise the dorsal column (DC) (constituted by axons of the primary sensory dorsal cells and dorsal root ganglion cells^43^,^44^) as well as the descending trigeminal tract, which is located next to the DC^45^, and processes of the cells of the dorsal population. In addition, some descending axons from the mesencephalic reticular nucleus (MRN) and the antero-lateral vagal group (ALV), which project to the medial (dorsal and/or ventral) spinal region^42^, may also descend in this region. Axons of the dorsal cells have been shown to have bad regenerative capacity^14^ and previous studies have shown that the projections from the ALV do not recover to normal levels even at 32 wpl, whereas axons of the MRN show good recovery at 32 wpl^16^. The regenerative capacity of dorsal root ganglion cells is unknown. The recovery of the number of processes at 2 wpl caudally, when the cells of the dorsal population are recovered in the rostral stump, suggests that these cells could sprout and/or branch extending their axons caudally, maybe to establish contacts to relay the descending drive. Although our results point to this reorganization, more anatomical and especially physiological studies are necessary to address this question more consistently.

The recovery of the number of dorsomedial profiles in the rostral stump at 2 wpl suggests that the processes of these cells sprout after the injury. Mechanisms of plasticity and sprouting in propriospinal circuits have been observed in many other vertebrates^5^,^6^,^7^,^9^,^10^,^29^,^46^, reviewed in ref. 11, including humans^8^. Interest in the plasticity of propriospinal circuits has been increasing recently with the observation of a high potential for structural and functional reorganization. Furthermore, plasticity of propriospinal networks could become a key mechanism for recovery of sensorimotor function after incomplete SCI in humans. In future work, it will be necessary to study the biological mechanisms underlying spinal circuit reorganization in lampreys (reviewed in ref. 11).

Our results strongly suggest that in regenerating vertebrates, like the sea lamprey, mechanisms of spinal reorganization and regeneration occur spontaneously in the glutamatergic system. Our study provides an accessible model system to decipher the molecular mechanisms underlying glutamatergic spinal circuit remodelling and regeneration and how these processes could be optimized for therapeutic interventions in mammals. Further, this work stresses the importance of studying the changes above the injury and not only below, as suggested before by other authors^47^ and also the necessity of increasing our understanding of the spinal circuits not only in lesioned but also in un-lesioned animals^48^,^49^.

## Methods

### Ethical statement

All experiments were approved by the Bioethics Committee at the University of Santiago de Compostela and the Consellería do Medio Rural e do Mar of the Xunta de Galicia (JLPV/IId) and were performed in accordance to European Union and Spanish guidelines on animal care and experimentation. During the experimental procedures, special effort was made to minimize animal suffering and to reduce the use of animals.

### Animals

Mature and developmentally stable larval sea lampreys, *Petromyzon marinus* L. (n = 45; between 80 and 156 mm in body length, 5 to 7 years of age), were used in the study. Most of the lampreys used in the study were between 90–125 mm long. One sample of 80 mm and another one 156 mm long were also used. In any case, lampreys used were randomly and homogeneously distributed between the different groups in terms of body length. Larval lampreys were collected from the river Ulla (Galicia, northwestern Spain), with permission from the Xunta de Galicia, and maintained in aerated fresh water aquaria at 15 °C with a bed of river sediment until their use in experimental procedures.

### Surgery

Larval lampreys were deeply anaesthetized by immersion in 0.1% tricainemethanesulphonate (MS-222; Sigma, St. Louis, MO) in Ringer solution (pH 7.4) of the following composition: 137 mMNaCl, 2.9 mMKCl, 2.1 mM CaCl_2_, 2 mM HEPES. Complete transection of the spinal cord at the level of the 5^th^ gill was performed as previously described^25^,^27^,^28^,^34^. Sham-operated controls were processed in the same way but only performing the surgical incision of the dorsal body wall without injuring the spinal cord.

### Tissue collection and processing

After the different recovery periods, control un-lesioned larvae (n = 6) and lesioned larvae with or without tracer [2 dpl (n = 5) and 1 (n = 3), 2 (n = 8), 4 (n = 5), 10 (n = 5) and 24 (n = 5) wpl] were deeply anaesthetized with 0.1% MS-222 in Ringer solution and killed by decapitation. The body region comprised between the 4^th^ and the 6^th^ gills was fixed by immersion in 5% glutaraldehyde and 1% sodium metabisulfite (MB) in 0.05 M Tris-buffered saline (TBS; pH 7.4) for 20 hours. After fixation, the tissue was washed and embedded in Neg 50^TM^ (Microm International GmbH, Walldorf, Germany), frozen in liquid nitrogen-cooled isopentane, sectioned on a cryostat in the transverse plane (14 μm thick) and mounted on Superfrost ^®^ Plus glass slides (Menzel, Braunschweig, Germany).

### Retrograde neuronal tracing

Tract-tracing experiments were performed to label axotomized neurons. Neurobiotin (NB, of 322.8 Da molecular weight; Vector, Burlingame, CA) was used as tracer. Larvae were deeply anesthetized with 0.1% MS-222 in Ringer solution and after a complete transection of the spinal cord at the level of the 5^th^ gill (see above), the tracer was recrystallized on the tip of a minute pin (size #000; 0.25 mm diameter) and applied into the rostral and caudal stumps of the cord. The animals were allowed to recover for 1 (n = 3) or 2 (n = 3) wpl in the same conditions as lesioned larvae without the tracer (see above).

### Immunofluorescence

For immunofluorescence, sections were pre-treated with 0.2% NaBH_4_ in deionized water for 45 minutes to quench autofluorescence. After being rinsed in TBS with 1% MB sections were incubated with a rabbit polyclonal anti-glutamate antibody (Immunosolution, Jesmond, Australia; 1:4500) or a mouse monoclonal anti-glutamate antibody (Swant, Bellinzona, Switzerland; 1:1000) in 0.05 M TBS with 1% MB for 3 days at 4 °C.Then, sections were incubated for 1 hour at room temperature with Cy3- conjugated goat anti-rabbit immunoglobulin (Millipore, Temecula, CA; 1:100) or fluorescein conjugated goat anti-mouse immunoglobulin (Millipore; 1:100). All antibodies were diluted in TBS (pH 7.4) containing 0.2% Triton X-100 and 15% normal goat serum. Traced samples were incubated at room temperature with DyLight 549 Streptavidin (Vector; 1:1000) diluted in TBS containing 0.3% Triton X-100 for 4 h. Nuclear counterstain was carried out by immersing the slides in 0.5 μg/mL bisbenzimide (Sigma) in TBS for 5 seconds. Slides were rinsed in TBS and distilled water and mounted with Mowiol.

### Antibodies

The polyclonal anti-glutamate antibody was raised in rabbit against a glutamate- glutaraldehyde-porcine thyroglobulin conjugate. The antibody has been tested by the supplier in sections of retina and cerebellum from various vertebrates, as well as in dot blot immunoassays with a variety of amino acid-protein conjugates. These include the standard 20 amino acids found in proteins, the non-protein amino acids D-serine, D-alanine and D-aspartate, GABA and the glycine containing tripeptide glutathione, which did not yield significant cross reactivity. This antibody was developed by Dr David V. Pow (University of Newcastle, New South Wales, Australia) and used in previous studies of the lamprey brain and spinal cord^25^,^30^,^41^,^50^,^51^. This antibody did not label any native protein band from the sea lamprey brain^51^.

The mouse monoclonal anti-glutamate antibody was first raised against glutaraldehyde-linked L-glutamate-bovine serum albumin (BSA) conjugate by P. Streit^52^, and the clone was made commercially available by Swant. The antibody has been characterized with respect to cross reactivity by antibody dilution experiments as well as by absorption experiments^53^. The same staining pattern was obtained with both monoclonal and polyclonal anti-glutamate antibodies in this study and in sections of the brain and retina (unpublished observations), and it does not differ from the pattern of expression of VGLUT as shown by *in situ* hybridization^30^,^41^,^51^.

### TUNEL assay

In order to detect DNA strand breaks as a sign of cell death, terminal deoxynucleotidyl transferase-mediated dUTP nick end labelling (TUNEL) staining was performed in sham-surgery control (n = 2), 1 (n = 3) and 2 dpl (n = 3) larvae. Animals were deeply anaesthetized with 0.1% MS-222 in Ringer and killed by decapitation. The body region comprised between the 4^th^ and the 6^th^ gills was fixed by immersion in 4% paraformaldehyde in 0.05 M TBS for 6 hours. After fixation, the tissue was washed and embedded in Neg 50^TM^ (Microm International GmbH), frozen in liquid nitrogen-cooled isopentane, sectioned on a cryostat in the transverse plane (14 μm thick) and mounted on Superfrost ^®^ Plus glass slides (Menzel). The TUNEL staining was performed according to the manufacturer’s protocol with minor modifications (*In situ* Cell Death Detection Kit, TMR red; Roche, Mannheim, Germany). Briefly, the slides were incubated in methanol for 15 minutes at −20 °C to permeabilize lipid membranes, followed by brief washes in PBS and another permeabilization in sodic citrate 0.01 M for 30 minutes at 70 °C. After several washes in PBS, the slides were incubated in the TUNEL reaction mix, containing the Label Solution (TMR red labelled nucleotides) and Enzyme Solution (Terminal deoxynucleotidyl transferase), for 90 minutes at RT. Slides were washed in PBS and distilled water and mounted with Mowiol. Positive controls were made by incubating some control section with recombinant DNAse I (400 U/ml; Roche) in PBS for 20 minutes at RT before the incubation in the reaction mix. Negative controls were made by incubating slides in the Label solution (without terminal transferase).

### Image acquisition

The sections were photographed and analysed by spectral confocal microscopy (models TCS-SP2 and SP5; Leica, Wetzlar, Germany). For the quantification of the number of glutamate-ir cells and fibres, one of each six consecutive sections between 150 to 650 μm rostral and caudal from the site of injury (region 2) were photographed. In control larvae, 10 sections at the level of the 5^th^ gill were photographed. For the quantification of double labelled glutamate-ir/neurobiotin cells, one of each four consecutive sections between 150 to 450 μm rostral and caudal from the site of injury (region 2a) were photographed. Photographs were taken at 20x magnification (with a 1.5x zoom lens) without changing the amplifier gain or the offset to avoid the introduction of experimental variability. Stacks of photographs were processed with LCS Lite (Leica) and Fiji (Image J, NIH, Bethesda, Maryland, USA) software to generate a Z projection of the stack and to compile a single tiff file of the photomicrograph. Contrast and brightness were minimally adjusted with Fiji software. Figure plates were generated and lettering was added using Adobe Photoshop CS4 (Adobe Systems, San José, CA, USA). Schematic drawings were made using Corel Suite X5 (Corel, Ottawa, Canada).

### Quantification of cells and fibres

Three types of glutamatergic cells were quantified: dorsal, lateral and CSFc cells. The cells were quantified manually by means of stereological counts of stacks of confocal microphotographs of spinal cord sections between 150 to 650 μm (region 2) rostral and caudal to the lesion site (only 1 out of 6 consecutive sections), as previously described^29^. Stereological counting was performed by disregarding the cells located in the first optical section of the confocal stack of each spinal cord section. The number of cells within the 500 μm between 150 and 650 μm from the site of injury (rostral and caudal) was inferred from the number of cells counted in each spinal section. Only one half of the cord was counted for each section. The mean number of cells was then calculated for each cell type and animal from the results of the sections analysed for the rostral and caudal spinal cord.

The number of glutamate-ir profiles (processes) was quantified using Fiji software, as previously described^50^. Briefly, the white matter of the spinal cord was divided into three different regions (DM, VM and LAT) (Fig. 1A). The glutamate-ir profiles were counted in each region in each spinal cord section. Again, only one half of the cord was analysed. A threshold was established to have the most accurate images, decrease background and increase edge and shape of the profiles. To establish this threshold value, ten random images with different profile densities were analysed before performing the actual quantifications and an optimal threshold value was selected. The same threshold was then used for all the photomicrographs. The mean number of positive profiles per section was then calculated for each region and animal (rostral and caudal).

The percentage of glutamate-ir cells labelled with neurobiotin was calculated using Image J by analysing 1 out of 4 consecutive sections rostral and caudal to the site of injury in three different individuals. Only cells that showed clear colocalization when going through the stack of confocal optical sections were considered. Imaging and quantification of cells and profiles were carried out by a blinded experimenter.

### Statistical analysis

Statistical analysis was carried out using Prism 6 (GraphPad software, La Jolla, CA). Data were presented as mean ± standard error of the mean (SEM). Normality of the data was determined by the Kolmogorov-Smirnov test and the homocedasticity was determined by the Brown-Forsythe test. The data that were shown to be normally distributed and homocedastic were analysed by a one-way ANOVA. Post-hoc Bonferroni’s multiple comparison test was used to compare pairs of data. The data that were not normally distributed were analysed by a Kruskal-Wallis test and post-hoc Dunn’s multiple comparisons test. The significance level was set at 0.05.

## Additional Information

**How to cite this article**: Fernández-López, B. *et al*. Anatomical recovery of the spinal glutamatergic system following a complete spinal cord injury in lampreys. *Sci. Rep.* **6**, 37786; doi: 10.1038/srep37786 (2016).

**Publisher's note:** Springer Nature remains neutral with regard to jurisdictional claims in published maps and institutional affiliations.
